# Experimental Evaluation of Herbivory on Live Plant Seedlings by the Earthworm *Lumbricus terrestris* L. in the Presence and Absence of Soil Surface Litter

**DOI:** 10.1371/journal.pone.0123465

**Published:** 2015-04-17

**Authors:** Johannes Kirchberger, Nico Eisenhauer, Wolfgang W. Weisser, Manfred Türke

**Affiliations:** 1 Terrestrial Ecology Research Group, Department of Ecology and Ecosystem Management, Technische Universität München, Hans-Carl-von-Carlowitz-Platz 2, D-85354 Freising, Germany; 2 German Centre for Integrative Biodiversity Research (iDiv) Halle-Jena-Leipzig, Deutscher Platz 5e, D-04103 Leipzig, Germany; 3 Institute for Biology, University of Leipzig, Johannisallee 21, D-04103 Leipzig, Germany; Estacion Experimental de Zonas Áridas (CSIC), SPAIN

## Abstract

**Background:**

Recent studies suggested that the earthworm *Lumbricus terrestris* might act as a seedling predator by ingesting emerging seedlings, and individuals were observed damaging fresh leaves of various plant species in the field. To evaluate the significance of herbivore behavior of *L*. *terrestris* for plant and earthworm performance we exposed 23- to 33-days-old seedlings of six plant species to earthworms in two microcosm experiments. Plants belonged to the three functional groups grasses, non-leguminous herbs, and legumes. Leaf damage, leaf mortality, the number of leaves as well as mortality and growth of seedlings were followed over a period of up to 26 days. In a subset of replicates 0.1 g of soil surface litter of each of the six plant species was provided and consumption was estimated regularly to determine potential feeding preferences of earthworms.

**Results:**

There was no difference in seedling growth, the number of live seedlings and dead leaves between treatments with or without worms. Fresh leaves were damaged eight times during the experiment, most likely by *L*. *terrestris*, with two direct observations of earthworms tearing off leaf parts. Another nine leaves were partly pulled into earthworm burrows. *Lumbricus terrestris* preferred to consume legume litter over litter of the other plant functional groups. Earthworms that consumed litter lost less weight than individuals that were provided with soil and live plants only, indicating that live plants are not a suitable substitute for litter in earthworm nutrition.

**Conclusion:**

Our results demonstrate that *L*. *terrestris* damages live plants; however, this behavior occurs only rarely. Pulling live plants into earthworm burrows might induce microbial decomposition of leaves to make them suitable for later consumption. Herbivory on plants beyond the initial seedling stage may only play a minor role in earthworm nutrition and has limited potential to influence plant growth.

## Introduction

Herbivory rates by invertebrates are often reported to be comparatively low, but effects depend on the system and group of organisms studied [1, 2]. Hulme (1996) [3], for instance, reported on a minor role of arthropods on the decrease of plant biomass and on plant survival, but found a stronger impact by mollusks. In contrast, insect outbreaks can lead to a significant loss of plant biomass [4]. In addition to rather weak direct effects of invertebrate herbivory, strong effects on plant community composition have often been reported to be mediated by indirect effects of herbivores altering the competition between plant species [2]. Whether some plant species benefit directly from herbivory remains controversial and has to be discussed with care [5].

Plant communities are strongly affected by detritivores in soil as those can change soil structure and recycle nutrients through consumption and egestion of organic material [6, 7]. Earthworms are often the most dominant taxon among soil biota, in terms of biomass and because of their functional relevance as decomposers. Moreover, they are considered as ecosystem engineers as they structure the soil for other organisms [8]. Earthworms do not only alter plant performance and plant community composition directly [9], they can also impact on the food choice and herbivory rates of generalist herbivores by changing the nutritional value of plant species [10].

The diet of earthworms mainly consists of dead plant tissue such as leaf litter, but animal dung, microorganisms, fungi, live or dead invertebrates and mineral soil fractions are also consumed [11]. Live plant material is also partly accepted by certain earthworm species, in particular, plant seeds [12] and probably living roots [11]. Freshly fallen leaves, however, are often not accepted before distasteful substances are degraded by microorganisms [11].

Recent observations have, however, questioned the assumption that earthworms mostly neglect fresh leaves and live plants. Eisenhauer et al. (2010) [13] found that the earthworm *Lumbricus terrestris* ingested emerging seedlings in the radicle and cotyledon stage from a number of species of legumes and grasses. The authors further concluded from natural ^15^N signatures in earthworm body tissue that seedlings were an important component of earthworm nutrition in their experiment. While these seedlings were very small and could be ingested whole by earthworms, another recent study based on video observations in the field emphasized the potential of *L*. *terrestris* to even act as a herbivore of adult plants by tearing off leaf fragments [14]. One of the video observations taken in the urban area of Sequim (Washington, USA) shows an individual of *L*. *terrestris* damaging a fresh leaf of common houseleek, *Sempervivum tectorum*. According to Griffith et al. (2013) [14], such scenarios could be observed repeatedly. To our knowledge such observations have not been documented in other studies, and thus this might be the first indication for this species being a potential aboveground herbivore.

In former studies on plant herbivory, earthworms such as *L*. *terrestris* were generally not considered, and, if earthworms do feed on live plants, their contribution to overall herbivory might have been overlooked and attributed to the feeding activities by other animal taxa. Stein et al. (2010) [15], for instance, investigated the impact of invertebrate herbivores in grasslands on plant species diversity, excluding invertebrate herbivores by applying pesticides against mollusks and insects on the soil surface. According to Edwards and Bohlen (1996) [16], less than 20 out of more than 200 pesticides are seriously toxic to earthworms, indicating that earthworms will probably not have been affected by the pesticides used in this and similar experiments. Hulme (1996) [3] applied pesticides against mollusks and additionally installed a fence around and on top of experimental plots to exclude mollusk, vertebrate and insect herbivores, but this likely also failed in excluding earthworms. If earthworms turn out to be significant herbivores, data of such experiments could possibly be misleading, because earthworms could have been involved in herbivory.

The observations of *L*. *terrestris* intentionally damaging fresh leaves documented in Griffith et al. (2013) [14] were carried out in stony garden areas with little plant litter available on the soil surface. Thus, earthworms might have been forced to feed on fresh leaves in order to survive, overestimating earthworm herbivory for situations where alternative food such as litter is available for the species.

A study was designed to further evaluate potential aboveground herbivore behavior in *L*. *terrestris* in the presence and absence of soil surface litter material. Using a microcosm experiment, we aimed to study the ecological significance of earthworm herbivory for plant performance and earthworm nutrition. Although we found that fresh leaves of several plants were damaged by earthworms or were partly pulled into earthworm burrows during the experiments, this happened very rarely. The results of our study do not support the hypothesis that *L*. *terrestris* is a significant herbivore.

## Methods

### Soil, earthworms, and plants

For pre-growing plants, keeping earthworms and for the experiments, we used clayey topsoil (pH 6.6) obtained from a meadow in Freising, Germany (coordinates N 48°24'04.60", E 11°44'00.35"). The meadow is the property of the Technische Universität München and soil was removed due to the construction of a parking area. No specific permissions were required for these locations and activities. Endangered or protected species were not harmed or affected in our study. The soil was sieved with a heavy-duty analytical sieve shaker (Analysette 18, Fritsch GmbH) with 8 mm mesh size and was heated to a temperature of 80°C in a drying oven (Memmert GmbH + Co. KG, models ULM700 and ULM800) for 24 h to kill seeds and soil invertebrates.

Individuals of *L*. *terrestris* were purchased from wurmwelten.de (Jasper Rimpau, Dassel, Germany). Earthworms were kept in plastic boxes with moistened soil at about 10°C in a fridge for two weeks to adapt to experimental soil conditions [17].

*Lumbricus terrestris* is a comparatively large anecic earthworm species with a body length of 120 to 300 mm. This species creates permanent or semi-permanent vertical burrows of up to one meter depth, reaching from the mineral horizon to the soil surface. Mean live body weight of the earthworms used in our experiments was 5.0 ± 0.18 g (mean ± SE; range = 3.32–7.89; n = 40). All individuals were clitellate. For comparison, mean weight of individuals of *L*. *terrestris* in the study of Eisenhauer et al. (2010) [13] on seedling predation was 4.05 ± 0.13 g.

Seeds were obtained from a seed supplier (Rieger-Hoffmann GmbH, Blaufelden-Raboldshausen, Germany). To be able to compare our results of earthworm herbivory to the preferences of earthworms for seeds, we chose the same plant species as used in Eisenhauer et al. (2009) [18] and partly also in the study by Eisenhauer et al. (2010) [13] on seed and seedling predation. Plants belonged to the three functional groups grasses, non-leguminous herbs, and legumes. Attractiveness of our study plants to herbivores was assessed previously in the Jena Experiment, a long-term plant diversity experiment, which differed considerably among the different plant species [1]; Herbivory rate, i.e. the percentage of leaf area damaged by sap sucking, leaf mining, rasping and chewing, of individual plant species growing in monocultures was calculated by dividing the area damaged by the original leaf area (both areas summed over all leaves in a sample; for details on the calculation see [1]). We used the herbs *Bellis perennis* L. (herbivory rate range 1.10–4.02%) and *Plantago lanceolata* L. (range 1.82–1.94%), the legumes *Medicago x varia* Mart. (range 2.07–6.43%) and *Trifolium repens* L. (range 6.18–7.44%) and the grasses *Phleum pratense* L. (range 0.30–0.48%), and *Poa trivialis* L. (range 0.99–1.37%).

### Microcosms

**Fig 1:**
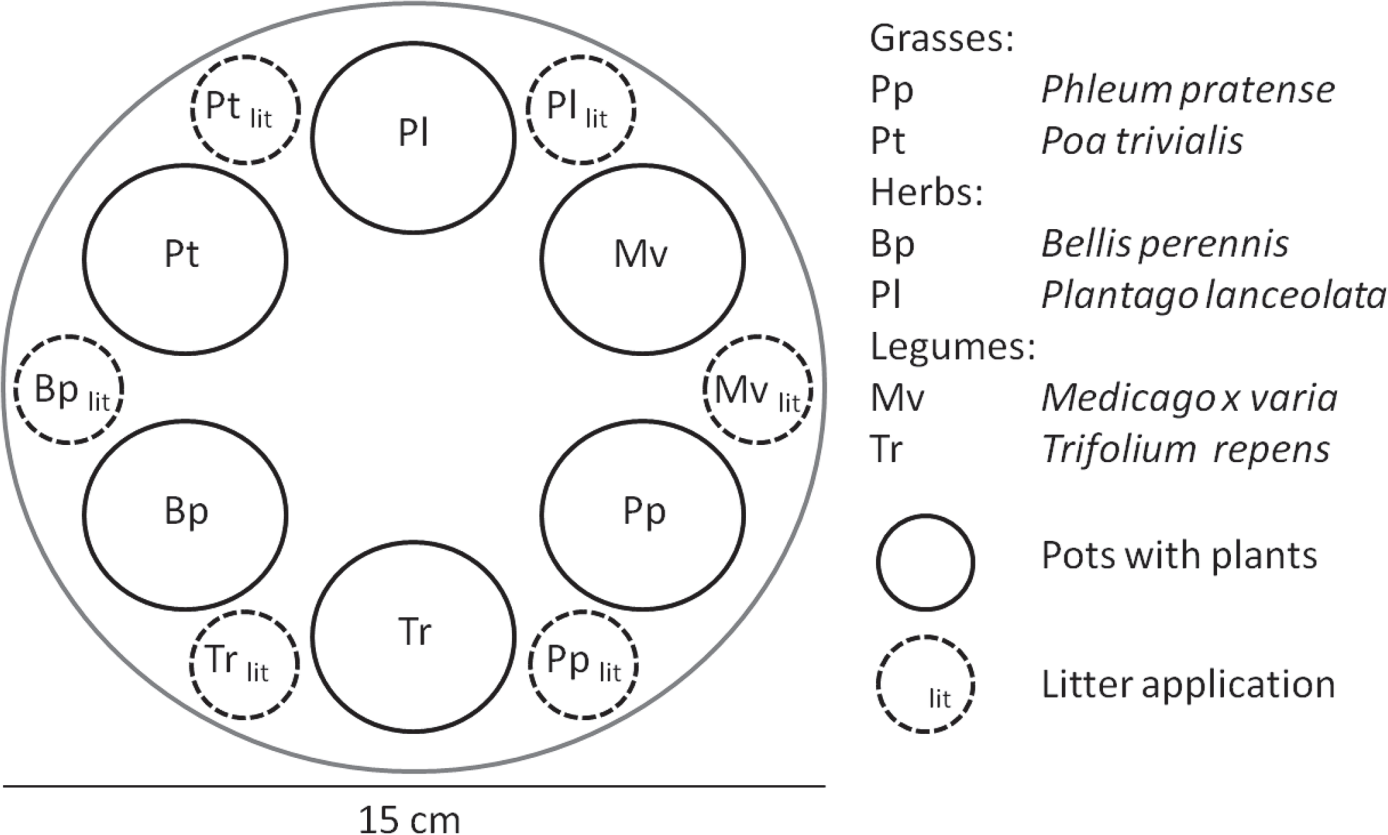
Schematic example of a microcosm (view from above). The six plant species were randomly arranged. Plant litter was placed on the soil surface next to the live plants of the same species in the respective treatments in the seedling mortality experiment.

Microcosms were built from rigid polyvinyl chloride tubes with a volume of 4.42 liter (diameter 15 cm, height 25 cm). Tubes were filled with soil up to the top. To prevent earthworms from escaping, the bottom of the tubes was closed with fly screen (mesh size 1.3 × 1.3 mm), and robust plastic foil was wrapped around the top (height of the plastic fence: 19 cm), which prevents earthworms from escaping the microcosms aboveground [17]. Thus, microcosms had not to be covered by a lid. Microcosms were set up in a greenhouse with regulated air exchange with the outside air and with automatic shading to prevent severe heating. On July 4, 2013 after weighing individuals of *L*. *terrestris* (accuracy 0.01 g), earthworms were transferred to microcosms of the respective treatments. Worms had four (herbivory experiment) or eight days (seedling mortality experiment) to adapt to the new environment before plants were added. Plastic pots with plants (diameter 3.5 cm, height 8 cm) were lowered almost entirely into the soil of each microcosm (Fig 1). Plants were isolated from the soil of the microcosm to exclude effects of earthworms on plant growth through changes in soil structure and nutrient availability. Control treatments without worms were arranged in between the earthworm treatments in order to balance microclimatic differences in the greenhouse chamber. Each microcosm was watered daily with about 4 l of tap water (less water was added if the soil was still wet) and plant pots with approximately 100 ml of water each. An excess of water could easily drain from microcosms as bottoms were only closed by fly screen. The average temperature on the soil surface of the microcosms was 26.3°C, ranging between 17 and 32°C. On July 22, 2013 microcosms were transferred to another greenhouse with facility for cooling because the average temperature increased to 28°C, a temperature which is lethal for *L*. *terrestris*. The average temperature in the new environment was 16.3°C, ranging between 14 and 22.5°C. At the end of the experiments, the soil of all microcosms was removed from tubes and examined for individuals of *L*. *terrestris*.

### Herbivory experiment

The aim of this experiment was to examine if *L*. *terrestris* damages fresh leaves when no soil surface litter material is provided and to test for potential preferences for certain plant species or functional groups. The experiment lasted 26 days and was conducted from July 8 to August 3, 2013. Twenty microcosms were provided with one individual of each of the six plant species, planted in seperate pots (diameter 3.5 cm, height 8 cm) and arranged circularly in the master pot, with the position of each species chosen randomly. At the beginning of the experiment seedlings were 33 days old, mean plant height was 7.5 cm (range 1.3–19.6 cm; depending on species) and plants had 8.4 leaves on average (range 3–23 leaves). Ten of the mesocosms were equipped with one individual of *L*. *terrestris* each (treatment) and ten without earthworms (control). Every two days we recorded survival of plants and the reason for death (e.g. consumption, desiccation), the number of live and dead leaves and the reason for leaf death, the number of leaves damaged and the area damaged (estimated in percent of leaf area missing), and the number of living leaves pulled into earthworm burrows. Furthermore, the height of each plant was measured at intervals of six days, starting on July 8 until August 1, 2013.

### Seedling mortality experiment

The objective of this experiment was to examine if *L*. *terrestris* kills seedlings when they are older than the cotyledon stage, either with or without soil surface litter material. Moreover, this experiment aimed to explore the feeding preferences of earthworms for seedlings and/or litter material of different plant species/ plant functional groups. This experiment lasted for 23 days and was conducted from July 12 to August 4, 2013. Forty microcosms were equipped with six different plant species, with five individuals planted in a seperate plastic pot per species. These pots were arranged circularly in the master pot, with the position of each species chosen randomly. At the beginning of the experiment seedlings were 23 days old and the smallest (mean height 4.0 cm, range 0.5–9.7 cm) and largest seedling (mean 6.6 cm, range 1.7–14 cm) of each species in each microcosm was measured. We established four treatments: (1) earthworm present, no soil surface litter (15 replicates); (2) no earthworm present, no soil surface litter (5 replicates); (3) earthworm present, with soil surface litter (15 replicates); (4) no earthworm present, with soil surface litter (5 replicates). In the respective treatments 0.1 g of oven-dried (80°C, 24h) shoot litter per plant species derived from 20–30 day old seedlings was placed on the soil next to the living plants of the same species (Fig 1). We recorded the survival of plants and the reason for plant death (e.g. consumption, desiccation) every two days. We recorded if leaves were damaged and if leaves were pulled into earthworm burrows. However, this was not done as intensively as in the herbivory experiment, because it was not the purpose of the seedling mortality experiment. The removal of soil surface litter material was estimated visually every two days in percentage cover from the initial area covered. We used a template of the size of the area originally covered by litter to estimate the proportion of litter removed [19]. Estimation of litter cover was always done by the same person.

### Statistical analysis

Statistical analyses were performed with R 3.1.2 [20]. All data were tested for normality and homogeneity of variance. Count data (number of leaves) were square-root transformed. In the herbivory experiment, we used an ANOVA to compare the initial number of leaves or plant height of plant individuals between treatments at the start of the experiment with the presence of earthworms and plant species as the fixed factors and with microcosms as the random factor as: model <- aov (y ~ worm treatment + plant species + Error(microcosm)). Further, we used an ANOVA to compare the number of leaves or plant height of plant individuals between treatments with the presence of earthworms, plant functional group, plant species and the days since the start of the experiment as the fixed factors and with plant functional groups nested within the date of each measurement nested within microcosms as the random factor and the initial number of leaves or initial plant height at the beginning of the experiment included as a covariate as: model <- aov (y ~ initial number of leaves OR initial plant height + worm treatment * functional group * plant species * days of experiment + Error(microcosm/date/functional group)). Further, we compared the sum of the number of leaves that died (desiccated and missing/disappeared for unknown reasons) over the course of the experiment of each plant individual between treatments as: model <- aov (y ~ worm treatment * functional group * plant species + Error(microcosm/functional group)). A similar model was calculated for missing leaves alone.

For the analysis of litter preference by earthworms in the seedling mortality experiment we used the 'persistence time' of litter of each plant species per microcosm, defined as the day on which at least 50% of the litter material of a species was consumed. As the experiment was ended after 23 days and litter of some species in several microcosms was not consumed until this time, we then calculated the proportion of days that litter persisted related to the total duration of the experiment. Proportions were arcsine square root transformed. Data was then analyzed using an ANOVA with litter persistence time as the dependent variable, worm treatment, plant functional group and plant species as the fixed factors and plant functional group nested in microcosm identity as the random factor as: model <- aov (y ~ worm treatment * functional group * plant species + Error(microcosm/functional group)).

To test whether survival of earthworms in both experiments combined differed among treatments, we used a generalized linear model with litter consumption (yes or no), body weight of worms at the start of the experiments (in g) and the experiment identity (herbivory or mortality) as the fixed factors and with a binomial error distribution as: model <- glm (y ~ litter consumption + body weight + experiment identity, family = binomial). To test whether the difference in body weight of earthworms in both experiments combined between the start and the end of the experiments differed between treatments, we used an ANOVA with litter consumption (yes or no) and the experiment identity (herbivory or mortality) as the fixed factors as: model <- aov (y ~ litter consumption + experiment identity). We performed tests with and without experiment identity included as a fixed factor and report results of the test with the lower Akaike information criterion (AIC) value.

Several earthworms presumably died during the experiments as we could not find an individual in the microcosm at the end of the experiments. The time of death of earthworms is unknown and might have happened early during the experiments and, thus, we excluded these replicates from all analyses following [17]. However, we performed all statistics also including data of replicates with dead earthworms. Results of these tests can be found in the S1 Statistics and are only mentioned in the main text if they differed from analyses without dead individuals.

Datasets of both, the herbivory and the seedling mortality experiment, can be found in the S1 Dataset and all statistical outputs are provided in the S1 Statistics.

## Results

### Herbivory experiment

Five replicates of the earthworm treatment had to be excluded from all statistical analyses as we could not find a worm in the microcosm at the end of the experiment [17]. No seedlings died over the course of the experiment. A total of three fresh leaves, one of *P*. *pratense* and two of *P*. *lanceolata*, were damaged, each with one third of leaf tissue removed. The missing part of one of the damaged leaves of *P*. *pratense* was found stuck in the soil and was most likely pulled into the ground but not ingested by an earthworm. Additionally, one leaf of *P*. *lanceolata* and one leaf of *P*. *pratense* were still attached to live plants but were partly pulled into the entrance of the earthworm burrow. All five incidences of earthworms attacking live plants were associated with two earthworm individuals.

**Fig 2:**
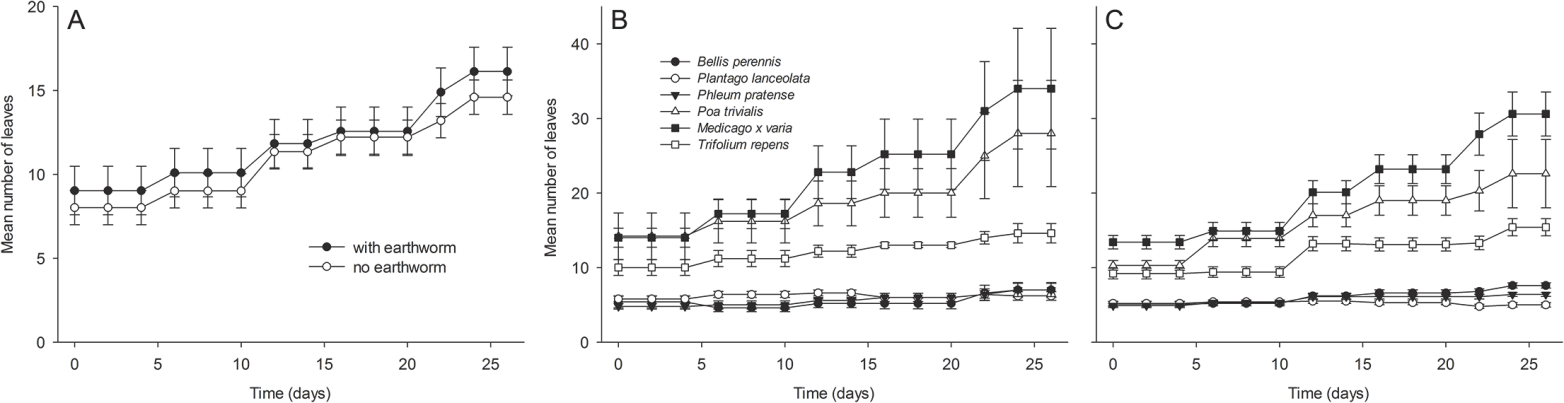
Number of leaves in microcosms with and without earthworms. Comparison of the number of living leaves (A) averaged over species in microcosms with and without *L*. *terrestris*, (B) among species in microcosms with *L*. *terrestris*, and (C) in microcosms without *L*. *terrestris*. Results are given as mean ± SE.

**Fig 3:**
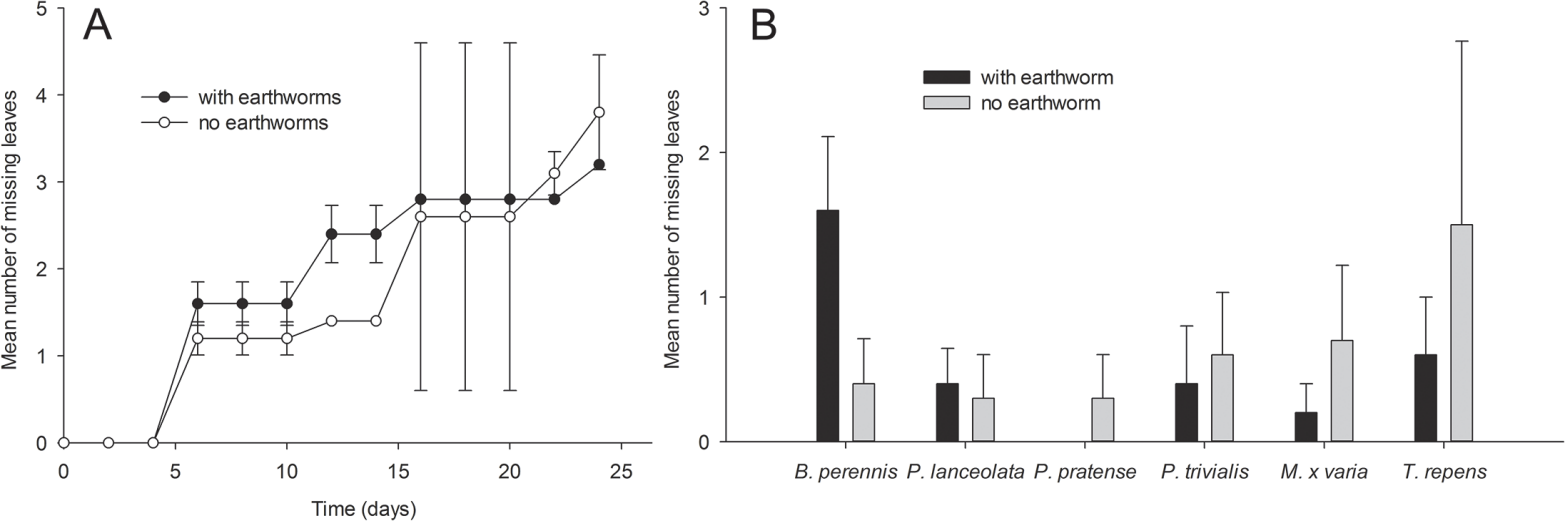
Number of missing leaves in microcosms with and without earthworms. Comparison of the number of missing leaves in (A) microcosms with and without *L*. *terrestris* averaged over species and (B) among the six different plant species. Results are given as mean ± SE.

The number of leaves per seedling and plant height did not differ significantly between treatments at the start of the experiment (S1 Statistics, S1.1 and S1.2). Results of an ANOVA comparing the number of leaves per seedling among treatments are shown in Table 1. The change in the number of leaves during the experiment in microcosms with (11.85 ± 1.31 mean ± SE per plant, taken all plant species together) and without *L*. *terrestris* (10.92 ± 0.92) did not differ significantly (Fig 2; Table 1; S1 Statistics, S1.3), but there was a significant interaction between the earthworm treatment and plant species (Fig 3; Table 1). For interpretation of the significant interaction, the number of leaves at the end of the experiment, after 26 days, are shown for earthworm treatments and controls in S1 Fig. There was also a significant interaction between the earthworm treatment and plant functional groups if replicates with dead earthworms were included (S1 Statistics, S1.3.2; p = 0.0161).

**Table 1:** ANOVA table for the analysis of the number of leaves and plant height in the herbivory experiment.

	Number of leaves			Plant height		
	DF	F-value	P-value	DF	F-value	P-value
Error: Microcosm						
Initial number of leaves/ Initial plant height	1	19.04	**<0.001**	1	10.60	**<0.01**
Treatment	1	0.02	0.897	1	0.15	0.710
Residuals	12			12		
Error: Microcosm:Date						
Days	1	1036.02	**<0.001**	1	223.01	**<0.001**
Treatment:Days	1	0.47	0.492	1	0.00	0.971
Residuals	193			58		
Error: Microcosm:Date:Functional group						
Initial number of leaves/ Initial plant height	1	4296.31	**<0.001**	1	1447.97	**<0.001**
Functional group	2	74.33	**<0.001**	2	52.58	**<0.001**
Treatment:Functional group	2	2.10	0.124	2	0.24	0.788
Functional group:Days	2	126.25	**<0.001**	2	23.58	**<0.001**
Treatment:Functional group:Days	2	0.38	0.685	2	0.115	0.892
Residuals	411			141		
Error: Within						
Initial number of leaves/ Initial plant height	1	4119.90	**<0.001**	1	197.31	**<0.001**
Plant species	3	50.44	**<0.001**	3	25.32	**<0.001**
Treatment:Plant species	3	8.54	**<0.001**	3	1.22	0.303
Plant species:Days	3	85.15	**<0.001**	3	4.68	**<0.01**
Treatment:Plant species:Days	3	1.67	0.173	3	0.27	0.849
Residuals	617			212		

ANOVA table of degrees of freedom (DF), F- and P-values for comparisons of the number of leaves per seedling and plant height according to treatment (earthworm present or not), the days since the start of the experiment, the three plant functional groups legumes ('plant species' *Medicago x varia*, *Trifolium repens*), non-leguminous herbs (*Bellis perennis*, *Plantago lanceolata*) and grasses (*Phleum pratense*, *Poa trivialis*) and with the initial number of leaves or initial plant height at the beginning of the experiment included as a covariate.

Significant effects (P<0.05) are given in bold.

In total, 83 leaves died during this experiment. While most of them disappeared for unknown reasons (54 missing leaves), the rest were mostly found to be desiccated. We found no herbivores other than earthworms on plants and in the greenhouse, which might be responsible for leaf consumption. There was no significant difference in the sum of the number of leaves that desiccated and of missing leaves in microcosms with (0.70 ± 0.17) and without *L*. *terrestris* (1.03 ± 0.23) (Table 2; S1 Statistics, S1.5), and also not if only missing leaves were considered in microcosms with (0.53 ± 0.23) and without *L*. *terrestris* (0.63 ± 0.16) (Fig 3; Table 2; S1 Statistics, S1.6).

**Table 2:** ANOVA table for the analysis of desiccated/ missing leaves in the herbivory experiment.

	Desiccated and disappeared leaves			Disappeared leaves		
	DF	F-value	P-value	DF	F-value	P-value
Error: Microcosm						
Treatment	1	0.63	0.443	1	0.00	0.992
Residuals	13			13		
Error: Microcosm:Functional group						
Functional group	2	1.45	0.253	2	0.63	0.542
Treatment:Functional group	2	2.03	0.152	2	1.75	0.194
Residuals	26			26		
Error: Within						
Plant species	3	1.29	0.290	3	1.82	0.159
Treatment:Plant species	3	0.55	0.652	3	0.68	0.572
Residuals	39			39		

ANOVA table of degrees of freedom (DF), F- and P-values for comparisons of the number of leaves that desiccated and leaves that disappeared for unknown reasons (= missing leaves) or of missing leaves alone according to treatment (earthworm present or not), plant functional group and plant species.

**Fig 4:**
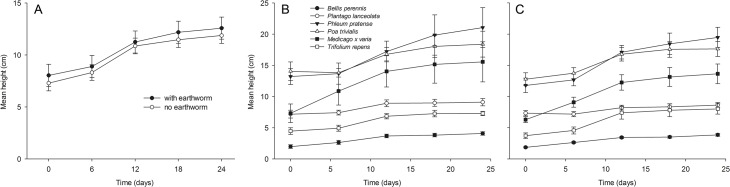
Plant height in microcosms with and without earthworms. Comparisons of (A) plant height averaged over species in microcosms with and without *L*. *terrestris*, (B) plant height among plant species averaged over microcosms with *L*. *terrestris*, and (C) plant height averaged over microcosms without *L*. *terrestris*. Results are given as mean ± SE.

Results of an ANOVA comparing plant height among treatments are shown in Table 1. The change in plant height during the experiment in microcosms with (10.59 ± 1.00 cm mean ± SE per plant, taken all plant species together) and without *L*. *terrestris* (9.96 ± 0.71 cm) did not differ significantly (Fig 4; Table 1; S1 Statistics, S1.4).

### Seedling mortality experiment

Three replicates of treatment 1 (earthworm present, no soil surface litter) and one replicate of treatment 3 (earthworm present, with soil surface litter) had to be excluded from the statistical analyses as we could not find an earthworm in the microcosms at the end of the experiment. Only nine out of 780 seedlings (1.15%) in microcosms with *L*. *terrestris* and six out of 300 seedlings (2%) in microcosms without *L*. *terrestris* died during the experiment, indicating no difference in seedling mortality between treatments (Fisher’s exact test, p = 0.382). The reason for the disappearance of seven of these plants was not clear: two of them were from microcosms without and five from microcosms with *L*. *terrestris*.

**Fig 5:**
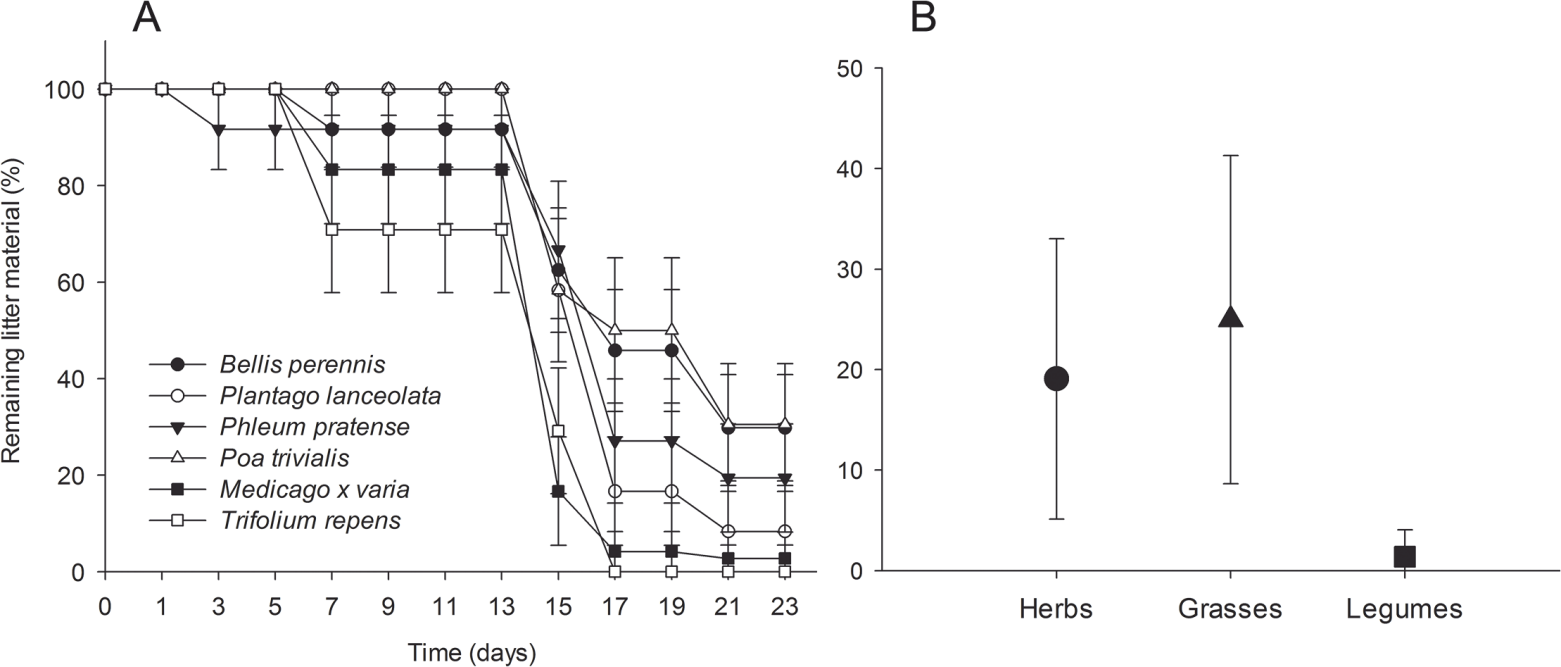
Decrease of soil surface litter consumed by earthworms. (A) Decrease of soil surface litter of different plant species in microcosms with *L*. *terrestris* (N = 12, results are given as mean ± SE) and (B) remaining litter of different plant functional groups after 23 days (results are given as mean ± 95%-confidence intervals).

Results of an ANOVA comparing litter persistence time among treatments are shown in Table 3. Litter material was not removed from the soil surface in the absence of earthworms (n = 5). By contrast, the amount of soil surface litter decreased over the course of the experiment in the presence of *L*. *terrestris* (n = 12, two of the 14 individuals did not feed on litter; Fig 5). On average, roughly half of the litter material (52%) was consumed after 15 days, and only 15% of the litter material remained after 23 days (end of the experiment). The persistence time of litter differed significantly between microcosms with and without *L*. *terrestris* (Table 3; S1 Statistics, S1.7).

**Table 3:** ANOVA table for the analysis of litter persistence time in the seedling mortality experiment.

	Litter persistence time		
	DF	F-value	P-value
Error: Microcosm			
Treatment	1	89.14	<0.001
Residuals	15		
Error: Microcosm:Functional group			
Functional group	2	9.36	<0.001
Treatment:Functional group	2	3.90	0.0312
Residuals	30		
Error: Within			
Plant species	3	0.75	0.526
Treatment:Plant species	3	0.31	0.815
Residuals	45		

ANOVA table of degrees of freedom (DF), F- and P-values for comparisons of the persistence time of litter according to treatment (earthworm present or not), plant functional group and plant species.

Significant effects (P<0.05) are given in bold.

At the end of the experiment *L*. *terrestris* had consumed most of the litter of the legumes *T*. *repens* and *M*. *x varia*, whereas more litter remained of species belonging to other plant functional groups (Fig 5). There was a significant difference in the persistence time of litter among plant functional groups and a significant interaction between earthworm treatment and plant functional groups (Table 3). This interaction was not significant if dead and non-feeding earthworms were included (S1 Statistics, S1.7.2). Legume litter was consumed faster than that of non-leguminous herbs and grasses (Fig 5). There was no significant difference among plant species within functional groups (Table 3).

In addition, we recorded damage on five leaves of living plants, only in earthworm treatments and in the absence of soil surface litter. One individual of *L*. *terrestris* was observed tearing off leaf fragments of two fresh leaves of *B*. *perennis* (damage 25 and 33% of leaf area, respectively), leaving the plant parts on the soil surface. In addition, we found more leaves being damaged without direct observation of earthworms tearing off leaf fragments, in particular, one leaf of *M*. *x varia* was damaged (33%) and two leaves of *T*. *repens* (50% each). The missing leaf fragments could not be found. Another four living leaves were pulled into the entrance of earthworm burrows in microcosms without soil surface litter (one *P*. *lanceolata*, one *T*. *repens*, two *B*. *perennis*) and three leaves in microcosms with soil surface litter (two *P*. *pratense*, one *P*. *lanceolata*). All twelve incidences of earthworms attacking live plants were associated with five earthworm individuals without access to litter material and by one individual with access to litter material. The single earthworm which fed on litter and attacked live plants did not consume any litter or attacked plants until July 25, but it had consumed litter of three plant species completely and had pulled three leaves of live plants into its burrow on July 27.

### Plants attacked by earthworms

Taken results of both experiments together, we noticed 17 incidences of earthworms attacking leaves of live plants, either tearing off leaf parts or pulling leaves into their burrows. These 17 incidences were distributed over seven out of 16 earthworm individuals without access to litter material (25 individuals including those which did not survive until the end of the experiments) and one out of 14 individuals (15 including dead earthworms) with access to litter material. Significantly more earthworms without access to litter material attacked plants than earthworms with access to litter material (Fisher’s exact test, p = 0.0395). If dead earthworm individuals were included in the analysis, the difference between groups did not remain significant (p = 0.219). The time of death of earthworms is, however, unknown and might have happened early during the experiments. No incidences of plants being attacked by earthworms were noticed in microcosms where no earthworms could be found at the end of the experiments.

### Body weight of earthworms

**Fig 6:**
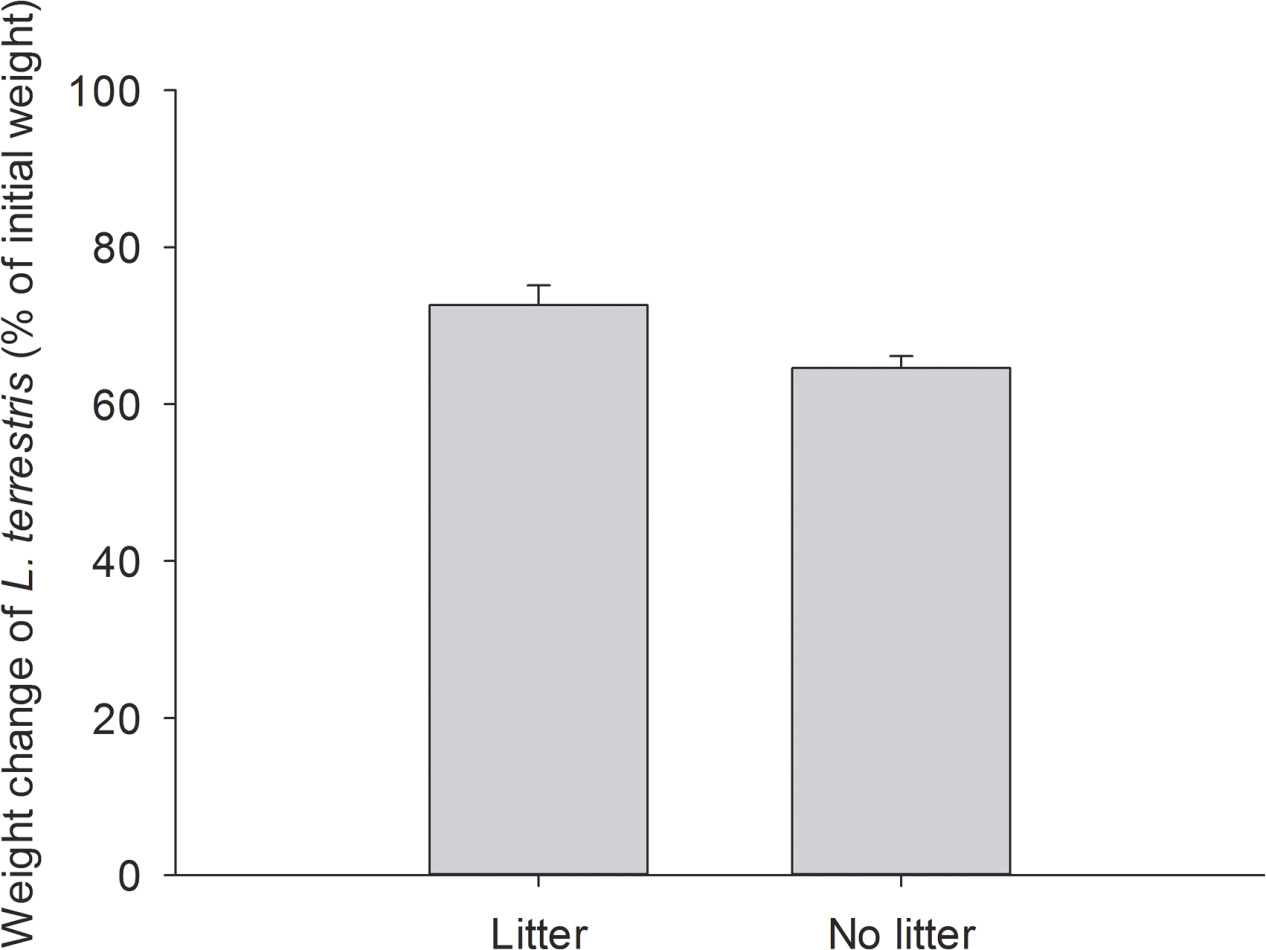
Weight change of earthworms feeding or not feeding on litter material. Comparison of weight change of individuals of *L*. *terrestris* which fed on litter material (n = 12, 0.1 g shoot litter per plant species was offered = 0.6 g in total) and individuals not feeding on litter material (n = 19) at the end of the experiments. Weight change differed significantly between groups (ANOVA, p = 0.017). Results are given as mean ± SE.

Body weight of earthworms at the start of the experiments did not differ between herbivory and seedling mortality experiment (ANOVA; F_1,38_ = 0.134, p = 0.717). Nine out of the 40 earthworms in both experiments were not found at the end of the experiment and most likely died during the experiments. The initial weight of individuals that were still alive after the experiment (5.19 ± 0.20 g mean ± SE, range 3.32–7.89, n = 31) was significantly higher than of individuals that died during the experiment (3.96 ± 0.58 g, range 3.49–6.45, n = 9) (S1 Statistics, S1.9; GLM; z = 2.24, p = 0.0253) indicating that mainly weak individuals died during the experiment. Eight of the nine earthworms died in microcosms without surface litter material, while only one individual died in microcosms with litter material, but neither litter consumption had a significant impact on survival (z = 1.18, p = 0.237) nor the type of experiment (z = 1.80, p = 0.0724). At the end of the experiments all living individuals of *L*. *terrestris* had lost weight. Individuals lost significantly less weight if litter material was consumed (28.92 ± 2.38%, range 5.24–39.08%, n = 12) than those which did not feed on litter material (35.39 ± 1.55%, range = 24.84–46.27%, n = 19) (Fig 6; ANOVA; F_1,29_ = 6.45, p = 0.0167).

## Discussion

The main objective of the present study was to investigate if the anecic earthworm *Lumbricus terrestris* is an aboveground herbivore as suggested by previous observations [13, 14]. Further, we wanted to test whether earthworms kill juvenile plants when feeding on them. We used a set of plant species from different functional groups to identify preferences of earthworms for leaves, seedlings, and soil surface litter material of certain species. As observations in Griffith et al. (2013) [14] of earthworms damaging live plants were made in garden areas where little litter material was available and thus herbivorous behavior might have been enforced, we tested whether earthworms damage plants under experimental conditions in the presence and absence of soil surface litter. In total, we noticed that eight leaves of different plants were damaged in our experiments, most likely by earthworms, and another nine leaves were pulled into earthworm burrows while still being attached to the live plants. We offered 810 seedlings to the 31 earthworms that survived until the end of our experiments and identified only 17 incidences of earthworms attacking leaves. Obviously, this is a very low number and leaf damage by earthworms may therefore be considered rare. Plant height and the number of leaves did not differ between microcosms with or without earthworms, but there was a significant interaction of earthworm treatment and plant species concerning the number of leaves (S1 Fig). Actually, there were slightly more leaves in earthworm treatments than in controls in the plant species *P*. *pratense*, *P*. *trivialis*, *M*. *x varia* and *P*. *lanceolata* and slightly less leaves in *B*. *perennis* and *T*. *repens*. Mortality of leaves (desiccated and missing) did not differ between treatments. Interpretation of these results is difficult. Incidences of earthworms attacking plants were rare and, thus, compensatory growth due to herbivory in some of the species in the earthworm treatment is unlikely. The number of missing leaves did also not differ between treatments with or without earthworms. Missing leaves might have often been cotyledons being shed by the plants and which decomposed quickly, but we did not separate between leaf types. Earthworms also had no significant impact on the survival of seedlings. Based on our results we conclude that earthworms are not important herbivores of plants having passed the cotyledon stage. In the following we discuss the potential ecological background of the behavior of earthworms damaging plants that we observed.

### Fresh plants in earthworm nutrition

*Lumbricus terrestris* primarily feeds on leaf litter, preferably mixed with some mineral soil, whereas most freshly fallen leaves are not accepted. Before earthworms feed on plant material it has to decay, which includes the softening of hard tissues, microbial degradation as well as leaching of feeding inhibitors [11, 16, 21]. Darwin (1882) [22], however, described in his seminal work on earthworms that also fresh leaves of a number of species are accepted by earthworms. Viable plant seeds are an exception as they are accepted as food by a number of earthworm species [18, 23]. First evidence that live plants are consumed by *L*. *terrestris* was provided by Eisenhauer et al. (2010) [13]. In this experiment, earthworms ingested small seedlings in the radicle or cotyledon stage with a maximum root length of 5 mm and a maximum shoot length of 6 mm. In such seedlings that still depend on stored reserves and that have limited photosynthetic area and root biomass, investment in defenses against herbivores is often low [24]. Young leaves and seedlings suffer strongly from herbivores as they lack structural carbohydrates, which contribute to leaf toughness and which increase during seedling development in many plants. Chemical defense is also often limited although secondary defense compounds can be at a high level in young seedlings of certain plants [24]. Our plants were juveniles but older than those in the study of Eisenhauer et al. (2010) [13]. Thus, we cannot rule out that increased plant defense and resistance might be reasons why earthworms rarely attacked seedlings in the present experiments. As the rhizosphere of plants in our experiments was isolated from the soil in microcosms, earthworms could not affect plant growth by changing soil properties. Thus, plant herbivore defense due to root herbivory or changes in soil conditions should also not have been altered in microcosms with earthworms compared to controls without earthworms.

On several occasions we observed that leaf fragments torn off by earthworms were left on the soil surface or in the soil of earthworm middens, and whole leaves still attached to plants were pulled into earthworm middens. This implies that earthworms might not consume the fresh plant material immediately. During decomposition plant secondary metabolites typically decline, which was found to increase palatability of certain plant species for earthworms [25]. Darwin (1882) [22] noticed that fresh leaves were pulled into earthworm middens and bathed in a fluid produced by earthworms, to which he referred to as 'extra-stomachal digestion' with the function to quickly kill and discolor leaves. Darwin also noticed, similar to our observations, that “the end of a leaf of *Triticum repens* (syn. *Elymus repens*), still attached to a growing plant, had been drawn into a burrow, and this part was dark brown and dead, whilst the rest of the leaf was fresh and green.” The behavior of earthworms leaving fresh leaves on or in the soil close to their permanent burrows might be a strategy to create palatable plant tissue to overcome food shortages. This assumption might be supported by the observation that significantly more earthworms attacked leaves in our experiments in treatments without soil surface litter material. Another indication that soil and live plants without supply of litter did not satisfy the nutritional needs of earthworms is that earthworms lost more body weight if they were not feeding on soil surface litter than individuals which fed on litter. The mortality and weight loss of worms might additionally have been enhanced by high temperatures during a period of our experiment (before microcosms could be shifted to a greenhouse with cooling facilities) as the mortality of *L*. *terrestris* increases with increasing temperature above 20°C, and earthworms deprived of food lose more weight at higher temperatures [26]. Because we used microcosms placed on the ground, they probably became warmer than soil in the field at the same air temperatures.

An alternative explanation to the phenomenon of earthworms damaging live plants is the following: earthworms plug up the entrances of their burrows with various objects and reopen them at night. Darwin (1882) [22] noticed that earthworms line their burrows with fine earth, little stones, twigs, petioles and use leaves of various plants for the entrance of their burrows, which might have also been the case for leaves and leaf fragments gathered by earthworms in our study. He further notes that these objects are probably not consumed by earthworms. In this case, earthworms do not damage plants for consumption at all and their activity cannot be considered as herbivory.

### Food preferences: litter vs. fresh plant tissue

*Lumbricus terrestris* fed on litter from all plant species with an average of 73% of the litter material consumed. Mean consumption differed among plant species, ranging from 60% (*P*. *trivialis*, *B*. *perennis*) to 86% (*T*. *repens*) of the initial amount of litter material. Earthworms preferred the legume litter over those of herbs and grasses. Litter of the two legumes *T*. *repens* (86%) and *Medicago x varia* (83%) were not only consumed or buried the most at the end of the experiment, but were also consumed faster than litter of the other species. Legumes are in symbiosis with nitrogen-fixing bacteria and often have comparatively high N concentrations in plant tissue and earthworms prefer leaf litter with a low C-to-N-ratio [11]. While Eisenhauer et al. (2010) [13] found that *L*. *terrestris* preferred legume seedlings at an early developmental stage over grass seedlings in food choice experiments, plants of the different functional groups were rarely damaged by earthworms in our experiments and also in comparable numbers. We recorded that 9 leaves of herbs, 4 leaves of grasses, and 4 leaves of legumes were damaged. The only species not damaged by earthworms was the grass species *P*. *trivialis* of which also the lowest amount of litter had been consumed at the end of the experiment. An exchange of nitrogen between plants of different species via roots was not possible in our experiments as plant species were planted in separate pots and were isolated from the soil of the microcosms.

## Conclusions

Results of the present study show that *L*. *terrestris* damages live plants. This, however, is likely to be a rare event and probably only happens if alternative food such as soil surface litter material is missing. Further experiments should investigate if *L*. *terrestris* consumes the torn off plant parts immediately or leaves them on or in the soil to decay and later consumption. *Lumbricus terrestris* preferred legume litter material over those of grasses and herbs, and data on earthworm weight change suggests that soil surface litter is a crucial component of the diet of this earthworm species which cannot be compensated for by herbivory. Our results on the minor biological significance of earthworm herbivory suggest that earlier herbivory studies ignoring earthworms did not introduce a major artefact. While herbivory on plants beyond the initial seedling stage may only play a minor role in earthworm nutrition and have limited potential to influence plant growth, earthworm effects on plants at the cotyledon stage needs further investigation.

## Supporting Information

S1 Dataset Meta-data and raw data of the herbivory and the seedling mortality experiment. (XLS)

S1 Fig Number of leaves per plant species at the end of the herbivory experiment. The number of leaves per plant species at the end of the herbivory experiment, after 26 days, in earthworm treatments and controls.
(TIF)

S1 Statistics Detailed results of statistical analyses. In the main text of the manuscript, tests were reported excluding replicates where no earthworms could be found in microcosms at the end of the experiments. We performed all statistics also on data including replicates with dead earthworms. The time of death of earthworms is, however, unknown and might have happened early during the experiment and in this case earthworms might have had only a small or no impact on the variables measured.
(DOCX)

## References

[1] Loranger H, Weisser WW, Ebeling A, Eggers T, De Luca E, Loranger J, et al Invertebrate herbivory increases along an experimental gradient of grassland plant diversity. Oecologia. 2014;174(1):183–93. 10.1007/s00442-013-2741-5 23907703

[2] Weisser WW, Siemann E. Insects and ecosystem function Berlin: Springer; 2004.

[3] Hulme PE. Herbivores and the performance of grassland plants: A comparison of arthropod, mollusc and rodent herbivory. J Ecol. 1996;84(1):43–51.

[4] Carson WP, Root RB. Herbivory and plant species coexistence: Community regulation by an outbreaking phytophagous insect. Ecol Monogr. 2000;70(1):73–99.

[5] Belsky AJ. Does herbivory benefit plants—A review of the evidence. Am Nat. 1986;127(6):870–92.

[6] Prather CM, Pelini SL, Laws A, Rivest E, Woltz M, Bloch CP, et al Invertebrates, ecosystem services and climate change. Biol Rev. 2013;88(2):327–48. 10.1111/brv.12002 23217156

[7] Wardle DA, Bardgett RD, Klironomos JN, Setälä H, van der Putten WH, Wall DH. Ecological linkages between aboveground and belowground biota. Science. 2004;304:1629–33. 15192218 10.1126/science.1094875

[8] Cole L, Bradford MA, Shaw PJA, Bardgett RD. The abundance, richness and functional role of soil meso- and macrofauna in temperate grassland—A case study. Appl Soil Ecol. 2006;33(2):186–98.

[9] Scheu S. Effects of earthworms on plant growth: Patterns and perspectives. Pedobiologia. 2003;47:846–56.

[10] Zaller JG, Parth M, Szunyogh I, Semmelrock I, Sochurek S, Pinheiro M, et al Herbivory of an invasive slug is affected by earthworms and the composition of plant communities. Bmc Ecol. 2013;13:20 10.1186/1472-6785-13-20 23668239 PMC3656784

[11] Curry JP, Schmidt O. The feeding ecology of earthworms—A review. Pedobiologia. 2007;50(6):463–77.

[12] Forey E, Barot S, Decaens T, Langlois E, Laossi K-R, Margerie P, et al Importance of earthworm-seed interactions for the composition and structure of plant communities: A review. Acta Oecol. 2011;37(6):594–603.

[13] Eisenhauer N, Butenschoen O, Radsick S, Scheu S. Earthworms as seedling predators: Importance of seeds and seedlings for earthworm nutrition. Soil Biol Biochem. 2010;42(8):1245–52.

[14] Griffith B, Türke M, Weisser WW, Eisenhauer N. Herbivore behavior in the anecic earthworm species *Lumbricus terrestris* L.? Eur J Soil Biol. 2013;55:62–5.

[15] Stein C, Unsicker SB, Kahmen A, Wagner M, Audorff V, Auge H, et al Impact of invertebrate herbivory in grasslands depends on plant species diversity. Ecology. 2010;91(6):1639–50. 20583706 10.1890/09-0600.1

[16] Edwards CA, Bohlen PJ. Biology and ecology of earthworms 3rd ed. London: Chapman and Hall; 1996.

[17] Fründ H-C, Butt K, Capowiez Y, Eisenhauer N, Emmerling C, Ernst G, et al Using earthworms as model organisms in the laboratory: Recommendations for experimental implementations. Pedobiologia. 2010;53(2):119–25.

[18] Eisenhauer N, Schuy M, Butenschoen O, Scheu S. Direct and indirect effects of endogeic earthworms on plant seeds. Pedobiologia. 2009;52(3):151–62.

[19] Eisenhauer N, Fisichelli NA, Frelich LE, Reich PB. Interactive effects of global warming and 'global worming' on the initial establishment of native and exotic herbaceous plant species. Oikos. 2012;121(7):1121–33.

[20] R Core Team. R: A language and environment for statistical computing Vienna, Austria: R Foundation for Statistical Computing; 2014; Available from: URL http://www.R-project.org/.

[21] Doube BM, Schmidt O, Killham K, Correll R. Influence of mineral soil on the palatability of organic matter for lumbricid earthworms: A simple food preference study. Soil Biol Biochem. 1997;29(3–4):569–75.

[22] Darwin C. The formation of vegetable mould: through the action of worms, with observations on their habits / by Charles Darwin; with illustrations. London: J. Murray; 1882.

[23] Shumway DL, Koide RT. Seed preferences of *Lumbricus terrestris* L. Appl Soil Ecol. 1994;1(1):11–5.

[24] Barton KE, Hanley ME. Seedling–herbivore interactions: Insights into plant defence and regeneration patterns. Ann Bot-London. 2013;112(4):643–50. 10.1093/aob/mct139 23925939 PMC3736773

[25] Schonholzer F, Kohli L, Hahn D, Daniel O, Goez C, Zeyer J. Effects of decomposition of leaves on bacterial biomass and on palatability to *Lumbricus terrestris* L. Soil Biol Biochem. 1998;30(13):1805–13.

[26] Daniel O, Kohli L, Bieri M. Weight gain and weight loss of the earthworm *Lumbricus terrestris* L at different temperatures and body weights. Soil Biol Biochem. 1996;28(9):1235–40.

