# Supplementary figures and images for: Experimental Evaluation of Herbivory on Live Plant Seedlings by the Earthworm Lumbricus terrestris L. in the Presence and Absence of Soil Surface Litter
Source: PLoS One. 2015 Apr 17;10(4):e0123465. doi: 10.1371/journal.pone.0123465 (PMC4401770; doi:10.1371/journal.pone.0123465)

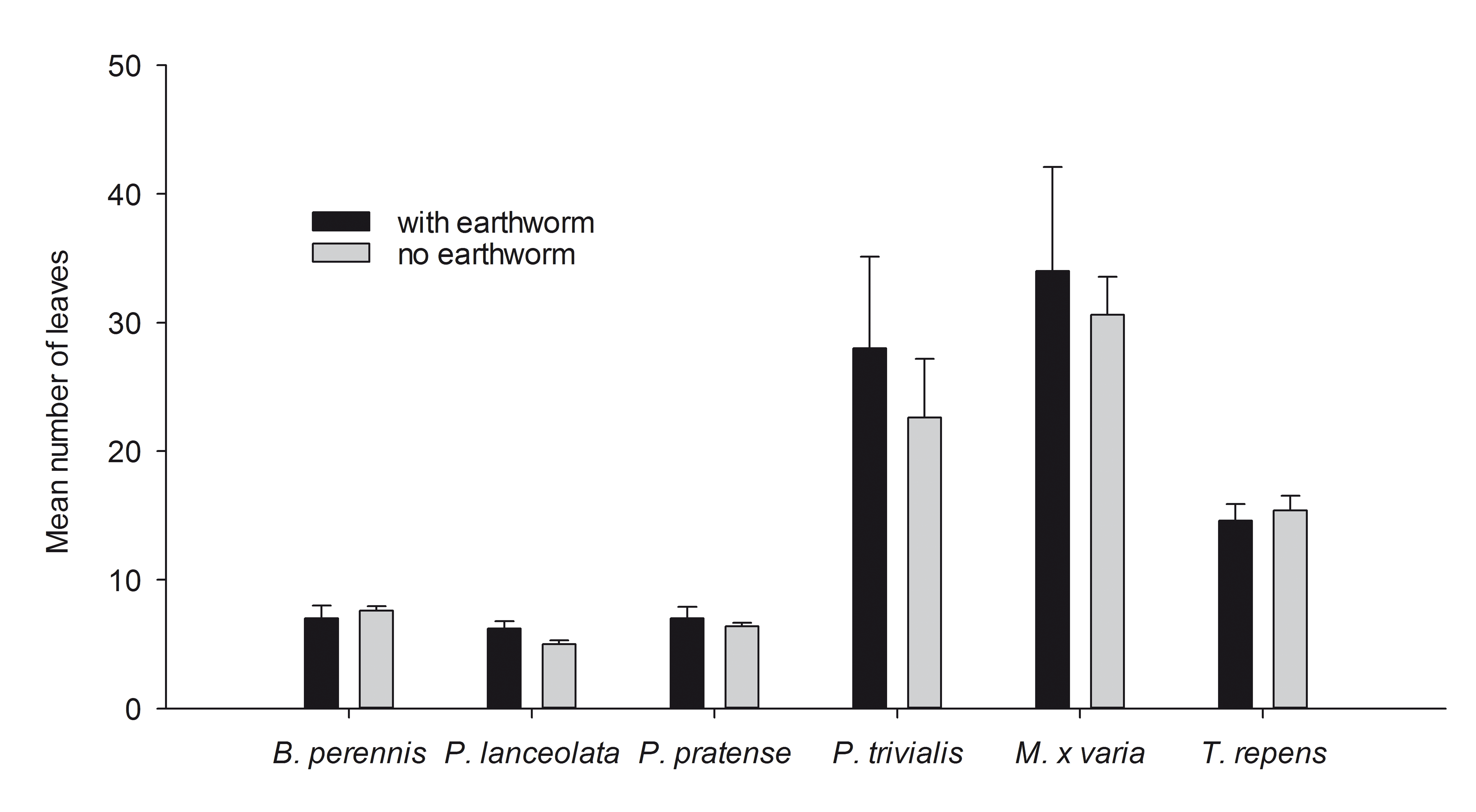

Supplement: S1 Fig — The number of leaves per plant species at the end of the herbivory experiment, after 26 days, in earthworm treatments and controls. (TIF) [file pone.0123465.s002.tif]
