# Supplementary material for: Experimental Evaluation of Herbivory on Live Plant Seedlings by the Earthworm Lumbricus terrestris L. in the Presence and Absence of Soil Surface Litter
Source: PLoS One. 2015 Apr 17;10(4):e0123465. doi: 10.1371/journal.pone.0123465 (PMC4401770; doi:10.1371/journal.pone.0123465)
Supplement: S1 Statistics — In the main text of the manuscript, tests were reported excluding replicates where no earthworms could be found in microcosms at the end of the experiments. We performed all statistics also on data including replicates with dead earthworms. The time of death of earthworms is, however, unknown and might have happened early during the experiment and in this case earthworms might have had only a small or no impact on the variables measured. (DOCX) [file pone.0123465.s003.docx]

**S1 Statistics: Statistical summary to Kirchberger et al.**

In the manuscript, tests were reported excluding replicates where no earthworms could be found in microcosms at the end of the experiments. We performed all statistics also including data with replicates with dead earthworms included, which are reported below. The time of death of earthworms is, however, unknown and might have happened early during the experiment and in this case earthworms might have had only a small or no impact on the variables measured.

Statistical analyses were performed with R version 3.1.2.

The factor 'Treatment' differentiates between microcosms with earthworms and controls without earthworms.

The factor 'Functional_group' includes the plant functional groups legumes ('Plant_species'*Medicago x varia, Trifoliumrepens*), non-leguminous herbs (*Bellis perennis, Plantagolanceolata*) and grasses (*Phleumpratense, Poatrivialis*).

The factor 'Experiment' differentiates between microcosms from the herbivory experiment and the mortality experiment.

The factor 'Days' indicates the number of days since the start of the experiment.

The factor 'Date' is the actual date of an assessment used to structure data as a random factor.

The factor 'Litter_consumed' differentiates between microcosms in which earthworms were feeding on surface litter material or not.

The factor 'Body_mass' is the live weight of earthworms at the start of the experiments.

**Herbivory experiment**

**S1.1 Number of leaves per seedling at the start of the experiment**

**S1.1.1 Model without replicates with dead earthworms**

aov(y~Treatment+Plant_species+Error(Microcosm))

Df Sum Sq Mean Sq F value Pr(>F)

Error: Microcosm

Treatment 1 0.3989 0.3989 4.258 0.0596 .

Residuals 13 1.2180 0.0937

Error: Within

Plant_species 5 30.161 6.032 42.68 <2e-16 ***

Residuals 70 9.893 0.141

**S1.1.2 Model including replicates with dead earthworms**

aov(y~Treatment+Plant_species+Error(Microcosm))

Df Sum Sq Mean Sq F value Pr(>F)

Error: Microcosm

Treatment 1 0.0118 0.01179 0.073 0.79

Residuals 18 2.9011 0.16117

Error: Within

Plant_species 5 37.74 7.548 54.6 <2e-16 ***

Residuals 95 13.13 0.138

**S1.2 Plant height of seedlings at the start of the experiment**

**S1.2.1 Model without replicates with dead earthworms**

aov(y~Treatment+Plant_species+Error(Microcosm))

Df Sum Sq Mean Sq F value Pr(>F)

Error: Microcosm

Treatment 1 11.00 11.001 2.993 0.107

Residuals 13 47.79 3.676

Error: Within

Plant_species 5 1501 300.21 57.1 <2e-16 ***

Residuals 70 368 5.26

**S1.2.2 Model including replicates with dead earthworms**

aov(y~Treatment+Plant_species+Error(Microcosm))

Df Sum Sq Mean Sq F value Pr(>F)

Error: Microcosm

Treatment 1 5.00 5.002 1.398 0.252

Residuals 18 64.41 3.578

Error: Within

Plant_species 5 1899.6 379.9 76.42 <2e-16 ***

Residuals 95 472.3 5.0

**S1.3Number of leaves per seedling over the course of the experiment**

**S1.3.1 Model without replicates with dead earthworms**

aov(y~Initial_number_of_leaves+Treatment*Functional_group*Plant_species*Days+Error(Microcosm/Date/Functional_group))

Df Sum Sq Mean Sq F value Pr(>F)

Error: Microcosm

Initial number of leaves 1 12.822 12.822 19.035 0.000924 ***

Treatment 1 0.012 0.012 0.018 0.896589

Residuals 13 18.081 1.391

Error: Microcosm:Date

Days 1 97.30 97.30 1036.016 <2e-16 ***

Treatment:Days 1 0.04 0.04 0.474 0.492

Residuals 193 18.13 0.09

Error: Microcosm:Date:Functional group

Initial number of leaves1 555.6 555.6 4296.313 <2e-16 ***

Functional_group 2 19.2 9.6 74.330 <2e-16 ***

Treatment:Functional_group

2 0.5 0.3 2.102 0.124

Functional group:Days 2 32.7 16.3 126.250 <2e-16 ***

Treatment:Functionalgroup:Days

2 0.1 0.0 0.379 0.685

Residuals 411 53.1 0.1

Error: Within

Initial number of leaves 1 476.4 476.4 4119.899 <2e-16 ***

Plant species 3 17.5 5.8 50.440 <2e-16 ***

Treatment:Plantspecies

3 3.0 1.0 8.541 1.45e-05 ***

Plant species:Days 3 29.5 9.8 85.151 <2e-16 ***

Treatment:Plantspecies:Days

3 0.6 0.2 1.665 0.173

Residuals 617 71.3 0.1

**S1.3.2 Model including replicates with dead earthworms**

aov(y~Initial_number_of_leaves+Initial_number_of_leaves+Treatment*Functional_group*Plant_species*Days+Error(Microcosm/Date/Functional_group))

Df Sum Sq Mean Sq F value Pr(>F)

Error: Microcosm

Initial number of leaves 1 27.819 27.819 32.53 2.59e-05 ***

Treatment 1 0.026 0.026 0.03 0.864

Residuals 17 14.539 0.855

Error: Microcosm:Date

Days 1 134.78 134.78 1488.026 <2e-16 ***

Treatment:Days 1 0.00 0.00 0.024 0.876

Residuals 258 23.37 0.09

Error: Microcosm:Date:Functional group

Initial number of leaves 1 731.2 731.2 5683.528 <2e-16 ***

Functional group 2 36.2 18.1 140.519 <2e-16 ***

Treatment:Functional group

2 1.1 0.5 4.160 0.0161 *

Functional group:Days 2 45.9 22.9 178.244 <2e-16 ***

Treatment:Functionalgroup:Days

2 0.5 0.3 2.096 0.1239

Residuals 551 70.9 0.1

Error: Within

Initial number of leaves 1 606.7 606.7 5319.393 <2e-16 ***

Plant species 3 24.6 8.2 71.814 <2e-16 ***

Treatment:Plant species

3 1.0 0.3 2.786 0.0398 *

Plant species:Days 3 38.4 12.8 112.102 <2e-16 ***

Treatment:Plantspecies:Days

3 0.2 0.1 0.617 0.6039

Residuals 827 94.3 0.1

**S1.4Plant height of seedling over the course of the experiment**

**S1.4.1 Model without replicates with dead earthworms**

aov(y~Initial_plant_height+Treatment*Functional_group*Plant_species*Days+Error(Microcosm/Date/Functional_group))

Df Sum Sq Mean Sq F value Pr(>F)

Error: Microcosm

Initial plant height 1 365.9 365.9 10.595 0.00689 **

Treatment 1 5.0 5.0 0.145 0.70998

Residuals 12 414.4 34.5

Error: Microcosm:Date

Days 1 1369.7 1369.7 223.006 <2e-16 ***

Treatment:Days 1 0.0 0.0 0.001 0.971

Residuals 58 356.2 6.1

Error: Microcosm:Date:Functionalgroup

Initial plant height 1 8374 8374 1447.966 <2e-16 ***

Functional group 2 608 304 52.576 <2e-16 ***

Treatment:Functionalgroup

2 3 1 0.239 0.788

Functional group:Days 2 273 136 23.575 1.47e-09 ***

Treatment:Functionalgroup:Days

2 1 1 0.115 0.892

Residuals 141 815 6

Error: Within

Initial plant height 1 1525.6 1525.6 197.309 <2e-16 ***

Plant species 3 587.3 195.8 25.319 4.85e-14 ***

Treatment:Plantspecies

3 28.3 9.4 1.220 0.30348

Plant species:Days 3 108.6 36.2 4.682 0.00345 **

Treatment:Plantspecies:Days

3 6.2 2.1 0.267 0.84908

Residuals 212 1639.2 7.7

**S1.4.2 Model including replicates with dead earthworms**

aov(y~Initial_plant_height+Treatment*Functional_group*Plant_species*Days+Error(Microcosm/Date/Functional_group))

Df Sum Sq Mean Sq F value Pr(>F)

Error: Microcosm

Initial plant height 1 519.4 519.4 11.137 0.0039 **

Treatment 1 5.0 5.0 0.108 0.7465

Residuals 17 792.8 46.6

Error: Microcosm:Date

Days 1 2066.4 2066.4 278.391 <2e-16 ***

Treatment:Days 1 7.8 7.8 1.054 0.308

Residuals 78 579.0 7.4

Error: Microcosm:Date:Functionalgroup

Initial plant height 1 11631 11631 1633.797 <2e-16 ***

Functional group 2 843 422 59.237 <2e-16 ***

Treatment:Functionalgroup

2 24 12 1.659 0.193

Functional group:Days 2 475 238 33.366 3.73e-13 ***

Treatment:Functionalgroup:Days

2 21 11 1.478 0.231

Residuals 191 1360 7

Error: Within

Initial plant height 1 2147.4 2147.4 251.881 <2e-16 ***

Plant species 3 729.9 243.3 28.538 3.53e-16 ***

Treatment:Plantspecies

3 35.8 11.9 1.401 0.243

Plant species:Days 3 206.7 68.9 8.080 3.48e-05 ***

Treatment:Plantspecies:Days

3 19.0 6.3 0.744 0.526

Residuals 287 2446.8 8.5

**S1.5The number of leaves that desiccated and were missing/disappeared for unknown reasons per seedling summed over the course of the experiment**

**S1.5.1 Model without replicates with dead earthworms**

aov(y~Treatment*Functional_group*Plant_species+Error(Microcosm/Functional_group))

Df Sum Sq Mean Sq F value Pr(>F)

Error: Microcosm

Treatment 1 0.348 0.3476 0.625 0.443

Residuals 13 7.231 0.5562

Error: Microcosm:Functional_group

Functional_group 2 1.580 0.7898 1.449 0.253

Treatment:Functional_group

2 2.209 1.1047 2.027 0.152

Residuals 26 14.172 0.5451

Error: Within

Plant_species 3 1.899 0.6330 1.293 0.290

Treatment:Plant_species

3 0.807 0.2689 0.549 0.652

Residuals 39 19.091 0.4895

**S1.5.2 Model including replicates with dead earthworms**

aov(y~Treatment*Functional_group*Plant_species+Error(Microcosm/Functional_group))

Df Sum Sq Mean Sq F value Pr(>F)

Error: Microcosm

Treatment 1 0.789 0.7889 1.749 0.203

Residuals 18 8.120 0.4511

Error: Microcosm:Functional_group

Functional_group 2 3.509 1.7543 3.055 0.0595 .

Treatment:Functional_group

2 2.675 1.3373 2.329 0.1119

Residuals 36 20.674 0.5743

Error: Within

Plant_species 3 2.670 0.8899 2.219 0.0964 .

Treatment:Plant_species

3 0.803 0.2675 0.667 0.5759

Residuals 54 21.657 0.4011

**S1.6The number of leaves that were missing/disappeared for unknown reasons per seedling summed over the course of the experiment**

**S1.6.1 Model without replicates with dead earthworms**

aov(y~Treatment*Functional_group*Plant_species+Error(Microcosm/Functional_group))

Df Sum Sq Mean Sq F value Pr(>F)

Error: Microcosm

Treatment 1 0.000 0.00 0 0.992

Residuals 13 5.459 0.42

Error: Microcosm:Functional_group

Functional_group 2 0.768 0.3840 0.628 0.542

Treatment:Functional_group

2 2.142 1.0708 1.750 0.194

Residuals 26 15.906 0.6118

Error: Within

Plant_species 3 1.687 0.5624 1.822 0.159

Treatment:Plant_species

3 0.625 0.2084 0.675 0.572

Residuals 39 12.038 0.3087

**S1.6.2 Model including replicates with dead earthworms**

aov(y~Treatment*Functional_group*Plant_species+Error(Microcosm/Functional_group))

Df Sum Sq Mean Sq F value Pr(>F)

Error: Microcosm

Treatment 1 0.116 0.1159 0.251 0.622

Residuals 18 8.303 0.4613

Error: Microcosm:Functional_group

Functional_group 2 0.834 0.4170 0.692 0.507

Treatment:Functional_group

2 1.553 0.7764 1.288 0.288

Residuals 36 21.696 0.6027

Error: Within

Plant_species 3 2.283 0.7609 2.860 0.0453 *

Treatment:Plant_species

3 0.363 0.1211 0.455 0.7148

Residuals 54 14.369 0.2661

**Seedling mortality experiment**

**S1.7Litter persistence time of surface litter material**

**S1.7.1 Model without replicates with dead earthworms and earthworms not feeding on litter**

aov(y~Treatment*Functional_group*Plant_species+Error(Microcosm/Functional_group))

Df Sum Sq Mean Sq F value Pr(>F)

Error: Microcosm

Treatment 1 6.343 6.343 89.14 1.06e-07 ***

Residuals 15 1.067 0.071

Error: Microcosm:Functional_group

Functional_group 2 0.6099 0.30494 9.358 0.000695 ***

Treatment:Functional_group

2 0.2541 0.12706 3.899 0.031241 *

Residuals 30 0.9776 0.03259

Error: Within

Plant_species 3 0.0897 0.02991 0.753 0.526

Treatment:Plant_species

3 0.0374 0.01246 0.314 0.815

Residuals 45 1.7865 0.03970

**S1.7.2 Model including replicates with dead earthworms and earthworms not feeding on litter**

aov(y~Treatment*Functional_group*Plant_species+Error(Microcosm/Functional_group))

Df Sum Sq Mean Sq F value Pr(>F)

Error: Microcosm

Treatment 1 4.313 4.313 14.43 0.00132 **

Residuals 18 5.381 0.299

Error: Microcosm:Functional_group

Functional_group 2 0.5184 0.25920 8.111 0.00124 **

Treatment:Functional_group

2 0.1728 0.08640 2.704 0.08054 .

Residuals 36 1.1504 0.03196

Error: Within

Plant_species 3 0.0763 0.02543 0.758 0.523

Treatment:Plant_species

3 0.0254 0.00848 0.253 0.859

Residuals 54 1.8120 0.03355

**Body weight of earthworms**

**S1.8Body weight of earthworms in both experiments at the start of the experiments (individuals which died in the course of the experiment are included)**

aov(Body_mass~Experiment)

Df Sum Sq Mean Sq F value Pr(>F)

Experiment 1 0.18 0.1771 0.134 0.717

Residuals 38 50.35 1.3249

**S1.9Survival of earthworms in both experiments at the start of the experiments**

**S1.9.1 'Experiment' included as fixed factor**

glm(y~Litter_consumed+Body_mass+Experiment, family = binomial)

Deviance Residuals:

Min 1Q Median 3Q Max

-2.09125 0.06688 0.26549 0.52352 1.60266

Coefficients:

Estimate Std. Error z value Pr(>|z|)

(Intercept) -7.2317 3.2336 -2.236 0.0253 *

Litter_consumed 1.5509 1.3101 1.184 0.2365

Body_mass 1.4418 0.6444 2.237 0.0253 *

Experiment 2.0224 1.1255 1.797 0.0724 .

(Dispersion parameter for binomial family taken to be 1)

Null deviance: 42.653 on 39 degrees of freedom

Residual deviance: 28.306 on 36 degrees of freedom

AIC: 36.306

**S1.9.2 'Experiment' not included as fixed factor**

glm(y~Litter_consumed+Body_mass, family = binomial)

Deviance Residuals:

Min 1Q Median 3Q Max

-2.3839 0.1020 0.3680 0.6016 1.6460

Coefficients:

Estimate Std. Error z value Pr(>|z|)

(Intercept) -5.127 2.727 -1.880 0.0601 .

Litter 2.341 1.208 1.938 0.0526 .

Body_mass 1.226 0.584 2.099 0.0358 *

(Dispersion parameter for binomial family taken to be 1)

Null deviance: 42.653 on 39 degrees of freedom

Residual deviance: 32.046 on 37 degrees of freedom

AIC: 38.046

**S1.10Difference in body weight of earthworms from both experiments between the start and the end of the experiments; dead individuals not included**

**S1.10.1 'Experiment' included as fixed factor**

aov(y~Litter_consumed+Experiment)

Df Sum Sq Mean Sq F value Pr(>F)

Litter_consumed 1 2.324 2.3239 6.286 0.0182 *

Experiment 1 0.101 0.1012 0.274 0.6050

Residuals 28 10.351 0.3697

AIC 61.96999

**S1.10.2 'Experiment' not included as fixed factor**

aov(y~Litter_consumed)

Df Sum Sq Mean Sq F value Pr(>F)

Litter_consumed 1 2.324 2.3239 6.448 0.0167 *

Residuals 29 10.452 0.3604

AIC 60.27152
